# Neurophysiological recordings from parietal areas of macaque brain during an instructed-delay reaching task

**DOI:** 10.1038/s41597-024-03479-7

**Published:** 2024-06-13

**Authors:** S. Diomedi, F. E. Vaccari, M. Gamberini, M. De Vitis, M. Filippini, P. Fattori

**Affiliations:** 1 Dept. of Biomedical and Neuromotor Sciences, University of Bologna, Bologna, Italy; 2 Alma Mater Research Institute for Human-Centered Artificial Intelligence, University of Bologna, Bologna, Italy

**Keywords:** Motor control, Sensorimotor processing, Neurophysiology

## Abstract

Facilitating data sharing in scientific research, especially in the domain of animal studies, holds immense value, particularly in mitigating distress and enhancing the efficiency of data collection. This study unveils a meticulously curated collection of neural activity data extracted from six electrophysiological datasets recorded from three parietal areas (V6A, PEc, PE) of two *Macaca fascicularis* during an instructed-delay foveated reaching task. This valuable resource is now accessible to the public, featuring spike timestamps, behavioural event timings and supplementary metadata, all presented alongside a comprehensive description of the encompassing structure. To enhance accessibility, data are stored as HDF5 files, a convenient format due to its flexible structure and the capability to attach diverse information to each hierarchical sub-level. To guarantee ready-to-use datasets, we also provide some MATLAB and Python code examples, enabling users to quickly familiarize themselves with the data structure.

## Background & Summary

Nowadays data sharing is the foundation of modern science, allowing for large-scale studies and the ability to replicate results^1^. The practice of exchanging data assumes ethical significance, especially in animal research. Beyond promoting collaboration and enabling scientists to build on one another’s work, it can help to reduce the need for a large number of animals used in research. This aligns with the guiding principles of the 3Rs in animal research, which aim to reduce, refine and replace the use of animals^2,3^.

In the field of Neuroscience, data sharing practices are shaped by the diverse rules set by journals. While some journals require authors to publicly share data on repositories, others may only request statements of data availability ‘upon reasonable request’. However, obtaining and understanding the shared data can be challenging for external researchers, as access is not always straightforward, and the dataset may lack clarity or documentation^4,5^. Indeed, requests for data are ignored in 14–41% of cases, while success is achieved in only 27–59% of instances^6,7^.

This work presents the results of an extensive data curation effort, driven by the aspiration to enhance the accessibility of our research data. Our work is based on consolidating multiple datasets from various papers^8–13^ into a single and easily accessible repository^14^, eliminating the need for researchers to request data. Within the manuscript, we detail the repository, providing essential insights for data reuse, with a clear and consistent terminology to describe the experimental task and the behavioral events recorded together with the neural activity. For the sake of completeness and transparency, we enriched the dataset with some experimental parameters not previously covered in other works and provided basic scripts to start interacting with the rich pool of information included in the datasets.

To guarantee the accessibility and the re-usability of our recordings, we adopted a file format (HDF5, Hierarchical Data Format 5) renowned for its convenience in data sharing and supported by a large open-source community^15,16^. Furthermore, HDF5 format does not require licenses and can be easily processes by both MATLAB and Python (the main programming languages in the Neuroscience community), as well as others.

**Fig. 1:**
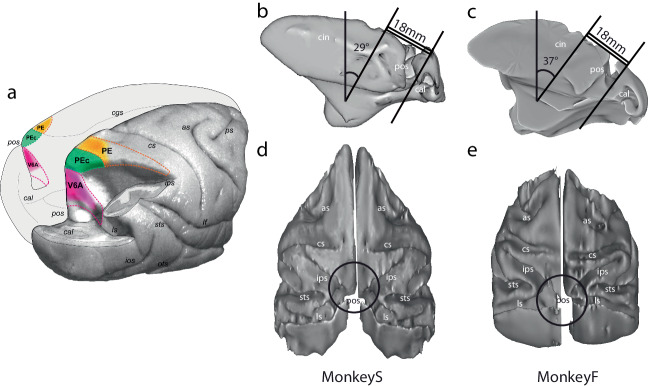
Anatomical locations of areas V6A, PEc, PE, and position of the recording chambers. (**a**) Posterolateral view of a right hemisphere and posteromesial view of a left hemisphere of M. fascicularis brain showing location and extent of areas V6A, PEc and PE of SPL (magenta, green and orange dashed lines, respectively). The right hemisphere is partially dissected to show the areas hidden in the parieto-occipital sulcus. Colored areas represent the reconstructions of the recording regions within these areas as a mean of four hemispheres from two animals (modified from^11^). (**b,c**) Mesial views of the three-dimensional reconstruction of the monkey brains, displaying the diameter and inclination of the recording chamber in each of the 2 monkeys. (**d,e**) Dorsal views of three-dimensional reconstructions of the monkey brains, displaying the location of the recording chamber in each of the 2 monkeys. The reconstructions were made using Caret software (http://brainvis.wustl.edu/wiki/index.php/Caret:About), starting from the histological sections^37,40^. The black circles provide an estimate of the chamber position and dimension. Abbreviations: as, arcuate sulcus; cal, calcarine sulcus; cs, central sulcus; ios, inferior occipital sulcus; ips, intraparietal sulcus; lf, lateral fissure; ls, lunate sulcus; ots, occipito-temporal sulcus; pos, parieto-occipital sulcus; ps, principal sulcus; sts, superior temporal sulcus.

The repository contains extracellularly recorded spiking activity from three areas (V6A, PEc and PE, Fig. 1a) located in the superior parietal lobule (SPL). The SPL is part of a network that combines information from motor and premotor areas with inputs from visual and somatosensory cortices. It plays a key role in in attention processing^17,18^, visuospatial perception^19^, and the preparation and execution of reaching and grasping movements^20–22^.

Within the SPL cortical convexity, area PE (Broadmann’s area 5) is located rostrally and contains neurons showing significant modulation during passive joint rotation, tactile stimulation, and active reaching movements involving both limbs^23,24^. More caudally, area PEc (Broadmann’s area 7) hosts visual, somatosensory and bimodal (somato-visual) neurons, alongside neurons that are modulated by arm reaching movements and gaze positions^25–28^.

The caudalmost area, V6A (Broadmann’s area 7), shares similar functional properties with PEc, but shows a higher proportion of visual neurons and fewer somatosensory neurons^21,29,30^. The cortical connections of these three areas are very extensive and reach pre-frontal, occipital areas but also many other parietal areas^31^.

**Fig. 2:**
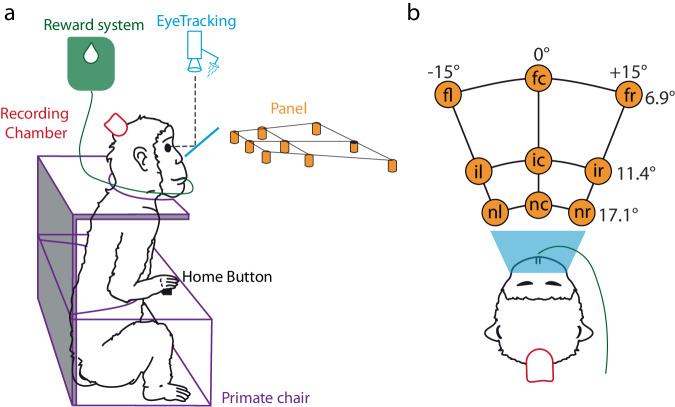
Experimental apparatus. (**a**) Lateral view of the experimental apparatus. Eye tracking system with cold mirror (Cyan), Reward system (Green), Recording chamber (Red), Primate chair (Purple), the Panel with the 9 targets (Orange). The Home Button embedded in the primate chair was used as the initial position for the reaching movements. (**b**) Top view of experimental apparatus. Spatial coordinates of targets are indicated as vergence (depth) and version (direction) angles in relation to the eyes. Abbreviations: fl, far left; fc, far central; fr, far right; il, intermediate left; ic, intermediate central; ir, intermediate right; nl, near left; nc, near central; nr, near right.

The recordings involved two monkeys (*Macaca fascicularis*, MonkeyS and MonkeyF), engaged in an instructed-delay foveated reaching task in the dark. Different from classic center-out reaching paradigms, our task introduces two significant modifications. First, our targets were arranged on a 2D horizontal plane at eye level (Fig. 2), allowing for the exploration of depth and amplitude components of reaching movements alongside different movement directions. Second, our task facilitates the replication of spontaneous, comfortable hand position close to the chest, providing a unique and naturalistic perspective on movement dynamics.

The repository comprises 6 datasets (3 areas for 2 animals), capturing the spiking activity of 285 V6A neurons (157 MonkeyS + 128 MonkeyF), 231 PEc neurons (134 MonkeyS + 97 MonkeyF), and 166 PE neurons (62 MonkeyS + 104 MonkeyF) that have been sorted and isolated.

The datasets here presented have been efficiently used in last works to functionally characterize individual neurons in the SPL, to model latent states and decode population activity^9,32^, but also to explain method of investigation^10,33^.

We firmly believe that sharing this dataset with the scientific community will hold high value not only for system neuroscientists but also for theoretical and computational researchers who are interested in studying the sensorimotor coordination of the efficient unfolding of arm actions which are orchestrated by our brain under physiological conditions.

## Methods

### Animal welfare statement

All procedures performed in this study are in accordance with the ethical standards of the University of Bologna. The study was performed in accordance with the guidelines of EU Directives (86/609/EEC; 2010/63/EU) and Italian national laws (D.L. 116-92, D.L. 26-2014) on the protection of animals used for scientific purposes. Protocols were approved by the Animal-Welfare Body of the University of Bologna and by the Ministry of Health, as requested by D.L. 26-2014, under the permit number N° 170/2015-PR, 19/03/2015 and N° 389/2019-PR, 20/05/2019.

The animals were housed in pairs in the animal facility in accordance with applicable Italian and European regulations. The facility provided the animals with natural as well as artificial light and enrichments including platforms and fresh fruits. Throughout the study, the animals’ health and behavioral welfare were carefully monitored by dedicated veterinarians, animal facility staff, and researchers who are all specialized in working with non-human primates and who are FELASA certified.

Non-human primate research is a small but essential part of neuroscience research. The scientists involved in this study recognise their responsibility to conduct high quality research while minimising any discomfort or distress experienced by the animals. Sharing the study data in a standard, open access format contributes to this commitment. It promotes transparency, accessibility, and optimal use of the data, adhering to the principles of the 3Rs and supporting international efforts to improve reporting and accessibility of biomedical research data.

### Animals and general procedures

Both animals were male long-tailed macaques (*Macaca fascicularis*). Monkey S was 4–6 years old and weighed between 3.9 and 4.9 kg during the experimental period; Monkey F was 5–8 years old and weighed between 3.7 and 4.0 kg. They were trained to perform an instructed-delay foveated reaching task. In the first phase of training, they became accustomed to sitting in a primate chair. Following this, the animals were brought into the laboratory to start task-specific training. The training sessions started with simplified versions of the task and gradually progressed until the entire task was learned. The training phase was considered complete when the monkeys achieved an acceptable performance.

Surgeries were performed aseptically using standard techniques, including appropriate perisurgical analgesia and monitoring to minimize potential suffering^34^. Before surgery, the animals were sedated with *ketamine hydrochloride* (i.m.) and then anaesthetized with *sodium pentobarbital* (i.v.) in order to implant the head holder and the recording chamber. Heart rate, respiratory rate, and body temperature were monitored. Body temperature was stabilized with a heating pad. The Veterinary Staff of the University of Bologna Veterinary clinic and the Neurosurgeons of the Neurological Hospital Bellaria contributed to the surgeries.

The head-holder consisted of a titanium headpost with a tripartite base to improve stability. It was located on the top of the skull at the same stereotaxic coordinates for both animals: mediolateral (ML): 0 mm; anteroposterior (AP): 19 mm anterior.

The recording chamber was implanted more anteriorly in Monkey S than in Monkey F (see Fig. 1b–e), using the following stereotaxic coordinates: for Monkey S mediolateral (ML): 0 mm; anteroposterior (AP): 13 mm posterior; the chamber was angled posteriorly with an inclination of 29° from the vertical axis; for Monkey F mediolateral (ML): 0 mm; anteroposterior (AP): 25 mm posterior; the chamber was angled posteriorly with an inclination of 37° from the vertical axis.

In both cases the center of the recording chamber was 10–12 mm anterior from the lambda suture.

### Daily routine

During the study, the animals had unrestricted access to food and fluid, except on the days of training and data recording. On these days, while in the lab, the animals received fluid rewards for each correctly performed trial. During a typical workday, the animal was taken out of its cage and placed in a primate chair where it was immediately weighed. Subsequently, it was brought to the laboratory for either a training or a recording session; the animal began to perform the task, and each successful trial was rewarded with water. During a recording session, the number of correct trials depended on the motivation of the animal, and in any case the session was terminated following the rhythm of the animal. At the end of the session, the animal was returned to its cage and its daily water intake was verified. If it was insufficient, additional water was supplemented directly in the cage. Access to food remained unaffected and was freely available at all times, independent of the animals’ performance in the experiments.

### Experimental setup

The experimental apparatus consisted of the following components: a monkey chair, an infrared eye-tracking system, a reaching panel, a reward system, electrophysiological recording hardware and software, experimental controller. The setup is sketched in Fig. 2.

The monkeys sat in a primate chair (Crist instruments, Hagerstown, MD, USA) equipped with a microswitch button to detect pressure from the hand (‘Home Button’ in Fig. 2a, 2.5 cm in diameter) placed in the center of the chair, 4 cm in front of the trunk of the animal. This button represented the starting position of each reaching movement, and pressing the button marked the beginning and end of each trial (see Task section). The chair was equipped with a bulkhead that allowed the arm not involved in the task to be retained. Monkeys performed the reaching movement with the contralateral arm in relation to the recording hemisphere.

Reaching movements were performed toward 1 of 9 target (multicolor LEDs green/red, 6 mm in diameter) starting from the home button (HB) position (Table 1). The nine targets were positioned on a horizontal panel at eye level, distributed across three main directions: left, central, and right. Along each direction, the distance between the three targets was chosen to reflect fixed increases in vergence. This design, which has been used in our laboratory, privilege targets placed on eye coordinates rather than Cartesian spatial coordinates, given the significant influence of eye position in the PPC^31,35^. Targets were arranged to ensure equal distances in eye coordinate space (version and vergence) rather than equal distances in Euclidean space.

**Table 1:** Spherical and Cartesian coordinates of target positions relative to the HB.

TARGET LABELS	Distance (cm) from HB	Laterality (°) relative to HB	Elevation (°) relative to HB	x (cm) relative to HB	y (cm) relative to HB	z (cm) relative to HB
near left	15.8	−15	52.4	−2.5	9.3	12.5
near central	16	0	51.3	0	10	12.5
near right	15.8	+15	52.4	+2.5	9.3	12.5
intermediate left	18.5	−15	42.4	−3.56	13.2	12.5
intermediate central	19.5	0	39.8	0	15	12.5
intermediate right	18.5	+15	42.4	3.56	13.2	12.5
far left	26.9	−15	27.7	−6.2	23	12.5
far central	27.9	0	26.6	0	25	12.5
far right	26.9	+15	27.7	6.2	23	12.5

Spherical coordinates of each target computed in a spherical coordinate system centred on the home button (origin) with Laterality axis aligned with the central targets. Cartesian coordinates of each target computed in a cartesian coordinate system centred on the home button (origin), with y axis aligned with the central targets (positive values moving away from the animal), x axis indicating laterality (positive values rightwards) and z axis indicating height (positive values upward).

Eye position was monitored using ISCAN ETL200 eye tracking system (sample frequency 100 Hz). The ISCAN cameras were placed above animal head and recorded the pupils indirectly thanks to a 45°-tilted hot mirror placed in front of animal eyes (represented in cyan in Fig. 2a,b). Eye position was controlled by an electronic window (4° x 4°) centred on the fixation target. If the monkey fixated outside this window during the required time intervals, the trial was aborted. At the beginning of each recording session, the monkey was required to perform a calibration task, consisting of the fixation of 10 targets on a fronto-parallel panel (for more detailed information on calibration task, see^11^).

The reward system consisted of a graduated cylinder containing water, connected to a solenoid valve that triggered the release of water through a feeding tube. The amount of water delivered after each correct trial was set in order to meet daily requirements.

In both animals, extracellular recordings were daily performed using a 5-channel multielectrode recording system (5-channel MiniMatrix; Thomas RECORDING GmbH, Giessen, Germany). The electrode signals were amplified (at a gain of 10,000) and filtered (bandpass between 0.5 and 5 kHz). The 5 channels were hardly used simultaneously, with utilization dependent on the ability to isolate stable neurons on individual channels. Action potentials in each channel were isolated with a waveform discriminator (Multi Spike Detector; Alpha Omega Engineering, Nazareth Illit, Israel) and were sampled at 100 kHz. The presentation of stimuli and the animal performance were monitored using custom software written in LabVIEW (National Instruments, Austin, US), as described previously^36^.

Histological reconstruction of electrode penetrations was performed following the procedures detailed in studies^34,37^ from our lab.

### Behavioural task sequence

The task was performed in complete darkness. A run of the experiment consisted of 10 correct trials for each target (90 correct trials in total). Targets were pseudo randomly interleaved, meaning that an error trial was always followed by a trial involving the same target whereas, upon correct trial completion, the target was chosen randomly. This approach ensured that the animals performed movements toward all targets for 10 trials each.

**Fig. 3:**
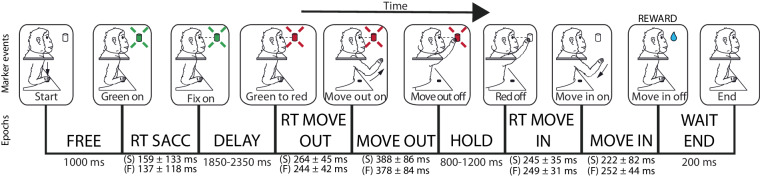
Task sequence. Top: task events from left to right: trial start (Start), target appearance (Green on), fixation onset (Fix on), go signal for the outward movement (Green to red), start of outward movement (Move out on), touch and beginning of the target holding (Move out off), go signal for the inward movement (Red off), release of the target and start of the inward movement (Move in on), end of the inward movement (Move in off), end of the trial (End). Bottom: placed between two adjacent event markers, there are the names of the task epochs and their duration. (S): MonkeyS, and (F): MonkeyF.

A trial began when the monkey pressed the HB. In the first part of each trial (FREE epoch, 1000 ms), the animal was free to look anywhere. When one of the 9 LEDs lit up green the monkey was required to perform the last saccade and start fixating the target (RT SACC). After the onset of fixation, the waiting interval began (DELAY). For each trial, the duration of this fixation was chosen randomly from two possible values (1850 and 2350 ms). During this epoch, a trial was aborted if the animal released the button. Then, the LED changed colour (from green to red), prompting the animal to move its arm and reach the target (RT MOVE OUT if the animal did not start moving within 1 second the trial was aborted. After the reaction time, the monkey released the HB and performed the reaching movement toward the target (MOVE OUT epoch). During the MOVE OUT period a trial was aborted if the reaching movement lasted longer than 1 second. Once the target had been reached, the animal was required to hold the reached position (HOLD epoch). The duration of the hold was randomly selected from two possible values (800 and 1200 ms). During HOLD a trial was aborted if the animal released the target before the switching off of the red light. Finally, the target turned off (Red off), allowing the animal to bring its hand back to the HB (RT MOVE IN and MOVE IN epochs), activating the reward system (Fig. 2). In the latter two epochs, a trial could be aborted if the animal needed more than 500 ms to start the backward movement or if the backward movement lasted longer than 1 second. After 200 ms (WAIT END epoch) the trial ended. Note that the animal maintained the fixation while the LED was turned on (from Green on to Red off events, Fig. 3), during this period, if the animal broke the fixation, the trial was aborted. The majority of epochs had durations that depended on the performance of the animal, with the exception of DELAY and HOLD as specified above and FREE and WAIT END which had a fixed duration.

## Data Records

In this section we will provide all the details that might be useful for the reuse of datasets.

The repository is publicly available via the German Neuroinfomatics Node (G-Node, http://www.g-node.org). The repository^14^ (~500 MB) contains datasets, metadata and some MATLAB/Python example scripts. We chose to format datasets and metadata in HDF5 files. This is a convenient format for sharing electrophysiological data for many reasons. First, it supports n-dimensional datasets, and each element in the dataset may itself be a complex object. It is a self-describing file format, meaning that data and descriptive metadata can be passed along in one file. Working with HDF5 is easy, because the dedicated software library runs on a range of computational platforms, from laptops to massively parallel systems, and implements a high-level API with C, C++, Fortran 90, and Java interfaces, as well as MATLAB and Python functions. Lastly, there is no limit to the number or size of data objects in each HDF5, providing the great flexibility needed for sharing experimental data.

Official tools that allow access to HDF5 files with no programming skills required can easily be found (https://www.hdfgroup.org/downloads/hdfview/).

We provide details and simple scripts to work with HDF5 files in MATLAB and Python languages, since they are widely used in the neuroscience community (see below). For MATLAB users additional information can be found at this link: https://it.mathworks.com/help/matlab/hdf5-files.html, while Python users can refer to the following page: https://docs.h5py.org/en/stable/.

We created 6 HDF5 files, 1 for each animal and area, named as follows:

1. MonkeyF_V6A_ reach9pos.h5
2. MonkeyF_PEc_ reach9pos.h5
3. MonkeyF_PE_ reach9pos.h5
4. MonkeyS_V6A_ reach9pos.h5
5. MonkeyS_PEc _reach9pos.h5
6. MonkeyS_PE_reach9pos.h5

In a few words, a HDF5 file is internally organized as a system of directories with folders and subfolders, files and labels attached to the folders. Each HDF5 hierarchical level (i.e. basically a folder / subfolder) is defined as ‘group’. Within each group can be stored the actual ‘datasets’. Labels defined ‘attributes’ can be attached to each group or each dataset containing metadata.

In describing the organization of our HDF5 files, we will use the term ‘condition’ (‘cond’). In the context of this repository, by ‘condition’ we mean the target of reaching. We adopt this general term in order to make the structure of our datasets generalisable for future data sharing of different tasks.

**Fig. 4:**
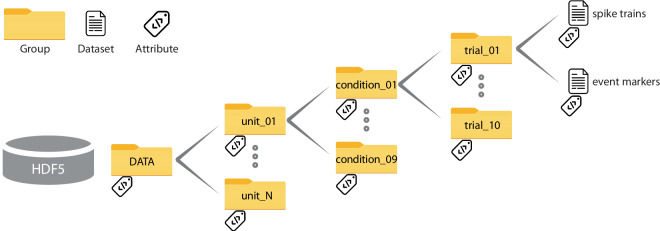
Schematic view of the HDF5 file organization. In each file, each group contains other groups up to the last hierarchical level in which datasets contain the actual data (similarly to the system of folders, subfolders, and files on a computer). Each group / dataset can be independently labelled with metadata (attribute).

The hierarchical organization of our 6 datasets is the following (Fig. 4):

Lv. 0: */***DATA**

*Lv. 1: /DATA/* **unit**

*Lv. 2: /DATA/unit*/**cond**

*Lv. 3: /DATA/unit/cond*/ **trial**

*Lv. 4: /DATA/unit/cond/trial*/**spike_trains**

*                 /DATA/unit/cond/trial*/**event_markers**

The first level (Lv. 1) contains a group for each unit recorded (unit_01, unit_02…). Similarly, the second level (Lv. 2) contains a group for each condition (target) used in the task (cond_01, cond_02…), and the third level (Lv. 3) contains a group for each trial recorded (trial_01, trial_02…). Note that the number of conditions is fixed for all datasets (9, corresponding with the 9 targets), whereas the number of units varies from one dataset to another. Also the number of trials can vary from one unit to another depending on how many trials could be successfully recorded. In some cases, a neuron’s spikes may have been lost before completing the task run.

The fourth and last level (Lv. 4) contains the actual data consisting of the timing (in ms) of each spike (*spike_trains*) or each behavioral event (*event_markers*) relative to the alignment marker. For instance, ‘*/DATA/unit_05/cond_03/trial_07/spike_trains’* will indicate the spiking activity recorded during the 7^th^ trial of the 3^rd^ condition of the 5^th^ unit in the dataset of interest. ‘*/DATA/unit_05/cond_03/trial_07/event_markers’* will indicate the corresponding behavioural events’ timing.

Metadata has been linked to groups and datasets as ‘attributes’ (Fig. 4).

Below is a list of the 16 attributes along with a short description. In brackets, the group/dataset to which the attribute is linked.

*Animal (DATA)*: animal’s name [‘MonkeyS’,‘MonkeyF’].

*Area (DATA)*: targeted area [‘V6A’,‘PEc’,‘PE’].

*Total neurons (DATA)*: total number of neurons for a specific dataset.

*Total conditions (DATA)*: total number of conditions (targets).

*Total event_markers (DATA)*: total number of event markers.

*Electrode id (unit)*: a letter identifier [‘A’,‘B’,‘C’,‘D’,‘E’,‘F’] indicating the recording electrode.

*Cito (unit)*: for V6A, it could be ‘V6Ad’ if the neuron was recorded from the dorsal part of V6A or ‘V6Av’ if it was recorded from the ventral part of V6A^34^. For the other areas this attribute shares the same information as the *Area* attribute.

*Hemisphere (unit)*: Identifier of the hemisphere from which the neuron was recorded, it could be ‘L’ for left and ‘R’ for right.

*Target label (cond)*: Labels of the targets [‘near left’, ‘near central’, ‘near right’, ‘intermediate left’, ‘intermediate central’, ‘intermediate right’, ‘far left’, ‘far central’, ‘far right’](see Fig. 2b).

*Laterality (cond)*: angle between the frontal (left-right) axis and the projections of the vector connecting initial position (HB) and target on the horizontal plane.

*Elevation (cond)*: angle between the horizontal plane and the vector connecting initial position (HB) and target.

*Distance (cond)*: Distance from the HB.

*3D cartesian coordinates (cond)*: Cartesian coordinates relative to the reference frame centred on the HB. X-axis corresponds to the frontal axis (positive rightward), Y-axis correspond to the sagittal axis (positive frontward), Z-axis corresponds to vertical axis (positive upward).

*Duration (trial)*: Duration of the trial in milliseconds.

*Number of spikes (spike_trains)*: Total number of action potentials recorded.

*Marker labels (event markers)*: Labels of the event markers [‘Start’,‘Green on’,‘Fix on’,‘Green to red’,‘Move out on’,‘Move out off’,‘Red off’,‘Move in on’,‘Move in off’,‘End’]. (see Fig. 3).

A summary of the attribute hierarchy is given in the following table (Table 2):

**Table 2:** Attribute hierarchy.

ATTRIBUTE	Type	levels					
		DATA	unit	cond	trial	spike_trains	event_markers
*Animal*	string	X					
*Area*	string	X					
*Total neurons*	double	X					
*Total conditions*	double	X					
*Total event markers*	double	X					
*Electrode id*	string		X				
*Cito*	string		X				
*Hemisphere*	string		X				
*Target label*	string			X			
*Azimuth*	double			X			
*Elevation*	double			X			
*Distance*	double			X			
*3D cartesian coordinates*	double			X			
*Duration*	double				X		
*Number of spikes*	double					X	
*Marker labels*	double						X

The recording apparatus stored the timing of the following events (event markers) during the task (Fig. 3) to track animal behaviour as the trial proceeded. The labels for each event marker (italic) stored in the HDF5 files are:

*Start*: monkey pressed the HB in the monkey chair marking the beginning of the trial.

*Green on*: one LED lit up in green, indicating the appearance of the target.

*Fix on*: the animal began to fixate the target that had turned lit up green (this event is detected by the eye-tracker).

*Green to red*: the LED changed colour from green to red, serving as the ‘go’ signal for movement toward the target.

*Move out on*: the HB was released (onset of the reaching movement toward the target).

*Move out off*: activation of the target microswitch (end of the reaching movement toward the target), confirming that the target had been reached.

*Red off*: the target light switched off (‘go’ signal for return movement toward HB).

*Move in on*: target release (onset of the reaching movement toward the HB).

*Move in off*: activation of the HB microswitch (end of the reaching movement toward the HB), indicating that the HB had been reached. This event coincided with the activation of the reward system.

*End*: 200 ms after *Move in off* trial ended.

**Fig. 5:**
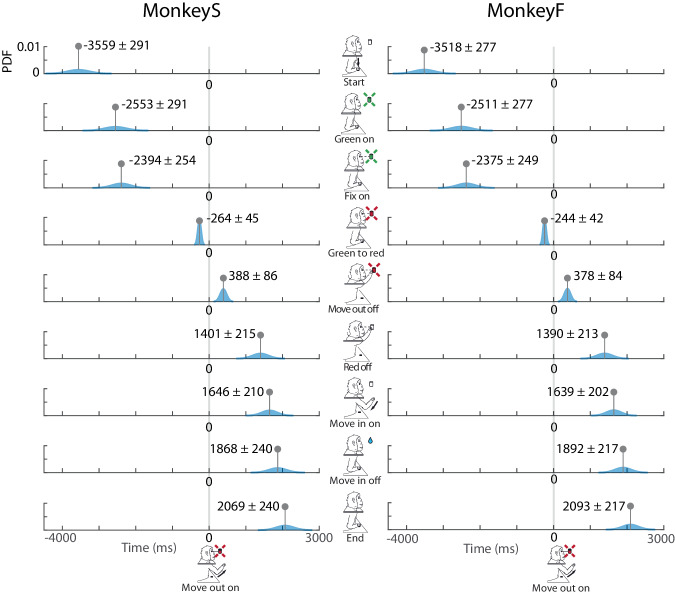
Event markers’ timing. In each row, the blue profiles represent the Gaussian probability distribution for each event timing relative to the alignment (Move out on, gray vertical line). Left column for MonkeyS, right column for MonkeyF.

In this repository, all the datasets published are arbitrarily aligned on the *Move out on* (reaching onset) event, meaning that for each trial the *Move out on* timestamp will be 0 and all the other timestamps (spikes and events) will be relative to this timescale. Users can easily realign the timestamps to the events of interest. Figure 5 shows the gaussian probability temporal distribution of each event markers previously described.

Note that, because of alignment, event markers that are temporally distant from movement onset exhibit increased temporal variability (Fig. 5).

The rection and movement time distribution shows a high degree of consistency between the two animals: reaction time of outward movement on average last for 254 ms while outward movement on average last for 383 ms, reaction time of inward movement on average last for 247 ms while outward movement on average last for 237 ms.

## Technical Validation

To prove the good quality of these datasets, the following checks were performed:

- Task epoch duration
- Inter Spike intervals

### Task epochs duration

We examined the consistency of event markers event timing with the proposed behavioral task. In particular, we calculated the mean duration of each trial epoch to assess its correctness with the task design and the physiological plausibility of time interval that depended on the monkey, such as reaction and movement times. Table 3 reports the average values obtained when pooling the 3 datasets for each animal. The saccade reaction time (RT SACC) durations for both animals were in line with previous reports (120–200 ms in monkeys^38^). Similarly, reaching movements were performed in 250–350 ms, consistent with previous findings (400 ms for movements toward objects at 12, 14, 18 cm^39^). Inward movements were significantly faster than outward ones, probably due to an effect of gravity or the simplicity of reaching the HB.

**Table 3:** Epoch duration.

Epochs	MonkeyS (mean ± std ms)	MonkeyF (mean ± std ms)
FREE	1007 ± 0.18 ms	1007 ± 0.22 ms
RT SACC	159 ± 133 ms	137 ± 118 ms
DELAY*	2130 ± 249 ms	2129 ± 249 ms
RT MOVE OUT	264 ± 45 ms	244 ± 42 ms
MOVE OUT	388 ± 86 ms	378 ± 84 ms
HOLD*	1013 ± 200 ms	1012 ± 200 ms
RT MOVE IN	245 ± 35 ms	249 ± 31 ms
MOVE IN	222 ± 82 ms	252 ± 44 ms
WAIT END	201 ± 0.13 ms	201 ± 0.14 ms

The mean and standard deviation of the various epochs defined by the task events were reported for each animal. Asterisks indicate epochs which duration was controlled by the task apparatus.

Note that, in some trials, the RT SACC interval can be extremely short (in the order of a few ms). This could happen whenever the monkey was already looking at the target before it lit up green, either by chance or because the current trial was preceded by an error trial and the system presented the same target again (thus, the monkey already knew the target position during the epoch FREE).

### Interspike Intervals

Computing the interspike intervals (ISIs) across the 6 datasets, we found that the smallest ISI was 0.38 ms and the longest was 5481.59 ms.

Additionally, we found that only a very small portion of the intervals fell within specific time ranges: 0.4% of the intervals fell between 6 seconds and 1 second; a significant majority (97.7%) were between 1 second and 1 ms; 1.9% were under 1 ms. These percentages are compatible with well isolated single cells and attest the quality of the data.

## Usage Notes

Recent publications from our group^32,33^ serve as examples of methods with which to analyse the neural data contained in these datasets, and are able to show examples of downstream processing steps. Note that to download the data files from the repository correctly, there are two options: (i) download a zip file containing data and code, directly from the official DOI webpage^14^ (‘Download Archive’ green button); (ii) download each data file manually by navigating to the ‘Data’ folder of the online repository webpage (https://gin.g-node.org/SDiomedi/SPL_single_units_reaching_task). In case of data reuse, please cite both this paper and the repository if possible; otherwise, cite only this paper.

**Fig. 6:**
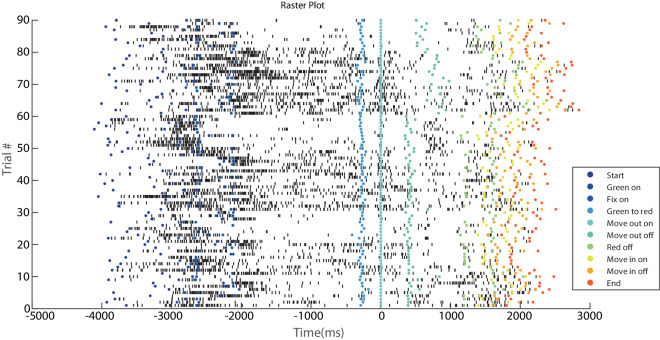
Raster plot example MonkeyS V6A unit_11. Example of raster plot of one neuron obtained with the script H5_raster. X-axis represents time and y-axis depicts the number of trials recorded. Each black line represents an action potential; each colored circle represents event markers, as in the legend.

**Fig. 7:**
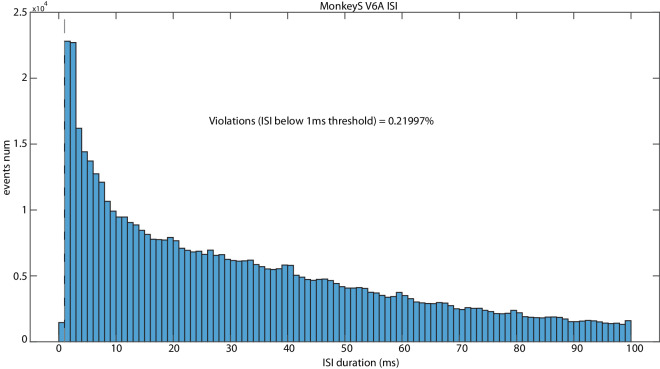
ISI plot example of MonkeyS V6A whole population using H5_ISI.m script. X-axis represents the distance between two consecutive spikes and y-axis shows the count of ISIs.

## Data Availability

Together with data, in the Repository users can find MATLAB and Python codes to start interacting with the datasets and obtain some simple plots. MATLAB code was written in MATLAB® R2022a (The MathWorks, Inc.). Python code was written using version 3.7.16. Required packages to run the provided scripts can be found in appropriate lists within the repository. We do not assure compatibility with older or newer MATLAB/python versions. We tested the execution speed of our scripts using the following hardware: a 13th Gen Intel(R) Core(TM) i7-1360P processor at 2.20 GHz, with 16.0 GB of installed RAM (15.7 GB usable). Specifically, the get_all_data_from_level_h5 function is particularly computationally intensive. On average, extracting data for a neuron (including all conditions and trials) takes 1.42 seconds using MATLAB and 0.18 seconds using Python (For example, extracting the entire MonkeyS_V6A_reach9pos.h5 dataset takes 260 seconds using MATLAB and 33 seconds using Python). Subsequent calculations and plotting, in the example scripts provided, require negligible time. The following examples derived from the MATLAB implementation, but Python functioning is identical. function [spikes, markers, all_strings] = get_all_data_from_level_h5(filename,str) % The function extracts the spikes and the markers from a h5 dataset % INPUT: %               filename = a string that identifies the h5 file from which extract data %               str = a string that identifies the group from which extract the data. The %               function automatically extracts every dataset of the group % OUTPUT: %               spikes = contains the extracted spike timing for each dataset %               markers = contains the extracted marker timing for each dataset %               all_strings = keeps track of the datasets extracted *Example script* **H5_raster** The script plots the neural and behavioral (event markers) data contained in an h5 file in the form of a raster plot (Fig. 6). Note that the User can change ‘str’ at the beginning of the script to plot: • all trials for a specific unit (‘/DATA/unit_01’, for example). • all trials for a specific unit AND condition (‘/DATA/unit_01/condition_01’, for example). • a specific trial for a specific unit AND condition (‘/DATA/unit_01/condition_01/trial_01’, for example) *Example script* **H5_ISI** The script calculates the Inter Spike Intervals (ISIs) and plots the results in a histogram. The proportion of ISIs violating a chosen threshold (normally, 1 ms) is also calculated and indicated in the histogram. Note that the User can change the variable ‘str’ at the beginning of the script to analyze all trials for a specific unit (all conditions), all trials for a specific unit and condition, or a specific trial for a specific unit AND condition combination (Fig. 7).
